# Gypenoside Protects Cardiomyocytes against Ischemia-Reperfusion Injury via the Inhibition of Mitogen-Activated Protein Kinase Mediated Nuclear Factor Kappa B Pathway *In Vitro* and *In Vivo*

**DOI:** 10.3389/fphar.2016.00148

**Published:** 2016-06-01

**Authors:** Haijie Yu, Liye Shi, Guoxian Qi, Shijie Zhao, Yuan Gao, Yuzhe Li

**Affiliations:** ^1^Department of Cardiology, The First Affiliated Hospital of China Medical UniversityShenyang, China; ^2^Department of Geriatrics, The First Affiliated Hospital of China Medical UniversityShenyang, China

**Keywords:** cardiomyocytes, gypenoside, I/R injury, MAPK, NF-κB

## Abstract

Gypenoside (GP) is the major effective component of *Gynostemma pentaphyllum* and has been shown to encompass a variety of pharmacological activities. In this study, we investigated whether GP is able to protect cardiomyocytes against injury myocardial ischemia–reperfusion (I/R) injury by using *in vitro* oxygen-glucose deprivation–reoxygenation (OGD/R) H9c2 cell model and *in vivo* myocardial I/R rat model. We found that GP pre-treatment alleviated the impairments on the cardiac structure and function in I/R injured rats. Moreover, pre-treatment with GP significantly inhibited IκB-α phosphorylation and nuclear factor (NF)-κB p65 subunit translocation into nuclei. GP and the MAPK pathway inhibitors also reduced the phosphorylation of ERK, JNK, and p38 *in vitro*. Specific inhibition of ERK, JNK, and p38 increased the cell viability of OGD/R injured cells. Taken together, our data demonstrated that GP protects cardiomyocytes against I/R injury by inhibiting NF-κB p65 activation *via* the MAPK signaling pathway both *in vitro* and *in vivo*. These findings suggest that GP may be a promising agent for the prevention or treatment of myocardial I/R injury.

## Introduction

As a predominant factor of heart injury, myocardial ischemia–reperfusion (I/R) is generally characterized by the deficient supply of blood flow to the myocardium, which leads to dramatic decrease in nutrients that are essential for energy generation. This disorder is always inevitable in that generation of I/R injury is associated with procedures used to re-establish blood flow to minimize heart damage due to severe myocardial ischemia (Kim et al., 2009; Ma et al., 2014), and such procedures are necessary for current therapies for coronary heart disease. These treatment modalities are effective in weakening acute myocardial ischemic injury and limiting myocardial infarction size, but they will themselves induce further cardiomyocytes death during the reperfusion process, namely I/R injury (Braunwald and Kloner, 1985; Piper et al., 1998; Yellon and Hausenloy, 2007). Unfortunately, although technologies and agents against myocardial disease are being rapidly developed, few effective strategies for prevention of myocardial I/R injury are available.

Ischemia–reperfusion injury can induce cell death or apoptosis, which is generally recognized to be initiated by reactive oxygen species (ROS) along with calcium overload and mitochondrial permeability transition (MPT) pore open (Wong et al., 2012). Moreover, various cytokines and Toll-like receptors are also released as part of the activation of the innate immune response in I/R injury (Frantz et al., 2005). The expression of ROS and innate immune factors leads to the downstream regulation of nuclear factor kappa B (NF-κB). NF-κB is a nuclear transcription factor regulating the immediate-early and stress response genes which are stimulated during acute inflammatory responses in various diseases, including ischemic pathology (Frantz et al., 2007; Wang et al., 2007). Previous evidence indicates the augmentation of NF-κB subunit p65 mediated inflammatory response in liver I/R injury model (Ramachandran et al., 2012). Several upstream pathways, such as MAPK and PI3K/Akt-related signal transduction, regulate the activation of NF-κB, i.e., phosphorylation of IκBα is initiated by PI3K/Akt phosphorylation, leading to the activation of NF-κB (Dilshara et al., 2014). NF-κB is also an important downstream target of MAPK signaling transduction in inflammatory and immune response (Baeuerle and Baltimore, 1996; Dai et al., 2011). It is reported that p38 MAPK (p38), c-Jun NH(2)- terminal kinase (JNK), and extracellular signal-regulated kinase (ERK) activation may up-regulate NF-κB by phosphorylation of IKK-β (Huang, 2008; Yeh et al., 2011; Slomiany and Slomiany, 2013). Moreover, p38 is also involved in the benzoquinone-mediated activation of NF-κB (Stokes and Winn, 2013) and an ERK-RhoA-NF-κB activation loop in breast cancer cells is also validated by Bist et al. (2015). Regarding I/R injury, the modulation of NF-κB by members of the mitogen-activated protein kinase (MAPK) signal transduction pathway is also verified (Chung et al., 2012; Himaya et al., 2012). Based on these studies, it is reasonable to conceive members of MAPK pathway as potential therapeutic targets for reducing cardiomyocytes loss due to myocardial I/R injury.

*Gynostemma pentaphyllum*, also known as ‘Jiaogulan,’ is a traditional medicine and herbal tea in Asian countries. The agent has been long time used as a medicine against chronic inflammation, hyperlipidemia, and liver diseases (Qin et al., 2012). Gypenoside (GP) is the predominant component of *Gynostemma pentaphyllum* and is capable of protecting tissues against inflammatory, tumor, and oxidant (Zhang G. et al., 2011; Zhang G.L. et al., 2011; Qin et al., 2012). Furthermore, therapeutic effects of GP on chronic hepatic injury in rat models have been reported (Zhang G.L. et al., 2011; Qin et al., 2012). However, few studies have investigated the effects of GP on myocardial I/R injury.

In the present study, the protective effects of GP against I/R injury were investigated by using *in vitro* oxygen-glucose deprivation–reoxygenation (OGD/R) H9c2 cell model and *in vivo* myocardial I/R rat model.

## Materials and Methods

### Materials

Gypenoside (purity > 99%) was purchased from Meilune Biotech (Catal. No. MB6716, Dalian, China) and dissolved in saline according to the manufacturer’s instruction. Based on the monograph of Chinese Pharmacopoeia Commission (1995), GP was obtained from the water extract of the aerial part of the *G. pentaphyllum* using chromatography method. **Supplementary Figure S1** shows the general structure for dammarane-type GPs (Aktan et al., 2003). Antibodies against NF-κB subunit p65 was purchased from Boster (Catal. No. BA0610. China). Antibodies against phosphorylated IκBα (p-IκBα), IκBα, p-ERK, ERK, p-JNK, JNK, p-p38, and p38 were purchased from Beijing Biosynthesis Biotechnology Co., LTD (Catal. No. bs-5515R, bs-1287R, bs-1522R, bs-2637R, bs-1640R, bs-10562R, bs-5477R, bs-0637R, Beijing, China). Antibody against β-actin was purchased from Santa Cruz Biotechnology, Inc. (Catal. No. sc-47778, Santa Cruz, CA, USA). MAPK pathway inhibitors U0126 (inhibitor of MEK1/MEK2), SP600125 (inhibitor of JNK), and SB203580 (inhibitor of p38) were purchased from Sigma–Aldrich (St Louis, MO, USA). All the material under current study is non-toxic to animals and cell cultures, including not only GP, but also all biologics and synthetics used for immunopharmacologic studies.

### Animal and Cell Cultures

H9c2 rat cardiac cell line was obtained from American Type Culture Collection (ATCC; Rockville, MD, USA) and incubated with DMEM/F-12 medium [10% (v/v) fetal calf serum and 1% (v/v) antibiotics mixture] in 95% air and 5% CO_2_ at 37°C. Eight-week-old male Wistar rats (weighing 240–260 g) were provided by Experimental Animal Center of China Medical University. Animals were raised at room temperature (20–25°C) with a constant humidity (55 ± 5%) with free access to food and water. All animal experiments were conducted in the accordance with the Institutional Animal Ethics Committee and Animal Care Guidelines for the Care and Use of Laboratory of Animals of Experimental Animal Center of China Medical University who governed the use of the animals.

### Cardiomyocytes I/R Injured Rat Models Establishment

Myocardial I/R injury model was induced based on previous study (Pfeffer et al., 1979) with some modifications. Briefly, the rats were anesthetized with pentobarbital sodium (50 mg/kg i.p.) before endotracheal intubation. After anesthesia, the animals were placed in a supine position and a lateral thoracotomy (1.5 cm incision between the third and fourth ribs) was performed to expose the left anterior descending coronary artery (LAD). A ligation using nylon suture was placed around the LAD at 3–5 mm for 45 min followed by 3 h of reperfusion. For rats in sham group, ddH_2_O was used instead of GP and rats were underwent the same surgical procedure without ligation. Ninety male Wistar rats were selected and randomly divided into five groups (18 for each group) and GP administration doses were employed according to study of Zhao et al. (2014) with some modifications (Wang et al., 2010): (1) I/R group, rats underwent I/R injury induction. (2) I/R + GPL group, rats were gavaged with 50 mg/kg body weight GP 1 h before model establishment. (3) I/R + GPM group, dose of GP was adjusted to 100 mg/kg body weight. (4) I/R + GPH group, dose of GP was adjusted to 200 mg/kg body weight. (5) Sham group. Of all the experimental animals in each group, six rats were used for hemodynamics parameters measurement and hematoxylin and eosin (H&E) staining, six ones were used for TTC staining and lactate dehydrogenase (LDH) and creatine kinase (CK) detection, and the left six were used for EMSA, ELISA, and Western blotting assay.

### OGD/R H9c2 Cell Model

For OGD treatment, H9c2 cells at log-growth stage (concentration adjusted to 5 × 10^4^/mL) were cultured in glucose-free DMEM medium in an atmosphere of 95% N_2_ and 5% CO_2_ at 37°C for 4 h. For reoxygenation, OGD cells were incubated with medium containing 4.5 mg/mL glucose and normal oxygen (21%) for 24 h. Five different treatments were set up and administration doses of GP were employed according to studies of Zhu et al. (2012): (1) control group, normal H9c2 cells. (2) OGD/R group. (3) OGD/R + GPL group, cells were incubated with 5 μM GP for 24 h before model establishment. (4) OGD/R + GPM group, dose of GP was adjusted to 10 μM. (5) OGD/R + GPH group, dose of GP was adjusted to 20 μM. Each treatment was represented by three replicates.

In addition, in *in vivo* experiment, the expressions of p-ERK, p-JNK, and p-p38 were all influenced by the treatment of GP. Based on these results, another three treatments with H9c2 cells were set up to reveal the possible mechanism involved in GP inhibiting NF-κB activity and administration doses of MAPK inhibitors on H9c2 was 10 μM according to previous studies (Aggeli et al., 2006; Yin et al., 2013): (6) OGD/R + U0126, 10 μM U0126 was added at the same time of GP administration. (7) OGD/R + SP600125, 10 μM SP600125 was added at the same time of GP administration. (8) OGD/R + SB203580, 10 μM SB203580 was added at the same time of GP administration. Each treatment was represented by three replicates.

### Hemodynamics Parameter Measurement

At the end of I/R procedure, left ventricular end systolic pressure (LVESP) and left ventricular end-diastolic pressure (LVEDP) were monitored with six awake rats in each group with a non-invasive blood pressure system (XBP 1000, Kent Scientific, Torrington, CT, USA) according to the manufacturer’s instruction. Briefly, the rats were fastened in a restrainer for a long period for the acclimation to the device, which was judged by the absence of struggling. The factional shortening (FS) was calculated by assuming a spherical left ventricular geometry with the algorithms of ultrasound system using Philips iE33 system (Philips Ultrasound, Bothell, WA, USA). All the parameters were represented by at least three replicates.

After the measurement of hemodynamics parameters, heart tissues of rats in different treatments were harvested and preserved in -80°C for H&E staining, TTC staining, and further analysis.

### Histopathological Staining

Hematoxylin and Eosin staining with ischemic penumbra part of heart tissue samples in each group was conducted for the observation of the histological changes of tissue samples in different groups according to the previous study (Mayer, 1896). The results were detected under a microscope at 200× magnification. Then the whole left ventricle of the heart of each sample was cut into five 2-mm transverse slices and the infarction areas were determined using TTC method. Tissue samples were incubated in 1% (TTC) in a 7.4 pH buffer for 10 min at 37°C to demarcate the infarction area: pale tissue was presumed to be infarcted. The infarcted area percentage of different samples was calculated using the Image-Pro Plus software.

### ELISA and EMSA

The levels of LDH and CK in serum samples were determined using ELISA assay kits (Catal. No. A032, A020-1, Nanjing Jiancheng Bioengineering Institute, Nanjing, China) according to the manufacturer’s instructions. The activities of NF-κB subunit p65 in different groups were measured using ELISA Kit (Catal. No. TE0001, Signosis, USA) according to the manufacturer’s instruction. The DNA-binding activity of NF-κB was quantified by EMSA as described previously (Hamid et al., 2009) using EMSA assay kit (Catal. No. SIDET001, Viagene, China).

### 3-(4,5-Dimethylthiazol-2-yl)-2,5-Diphenyltetrazolium Bromide (MTT) Test

Upon completion of the 24 h reoxygenation, MTT assay was performed to determine the viability of H9c2 cells in different groups. Briefly, 50 μL exponentially growing cells (2 × 10^5^ cells/mL) were seeded into a 96-well plate in triplicate. Then 5 mg/mL MTT was added to each well and incubated for 4 h at 37°C. The OD values at 490 nm in different wells were recorded using a Microplate Reader.

### Immunofluorescent Microscopy

The nuclear translocation of NF-κB subunit p65 was detected with immunofluorescent microscopy. Briefly, the treated cells were seeded into 24-well chambers, washed with PBS, fixed with 4% paraformaldehyde for 15 min. Then the cells were permeabilized with 0.5% Triton X-100 for 30 min. After being washed with PBS for three cycles, the cells were blocked in 10% goat serum for 15 min. Primary rabbit polyclonal antibodies (1:200) to NF-κB p65 was then added and the cells were incubated overnight at 4°C in 1% goat serum. Staining was performed by incubating the cells with fluorescein isothiocyanate secondary antibody for 1 h. After incubation with the secondary antibody, cells were washed and then stained with 4,6-diamino-2-phenyl indole (DAPI) for 5 min at room temperature. After extensive washing with PBS, the slides were fixed and imaged with the fluorescent microscopy at 400× magnification.

### Western Blotting

The total proteins were extracted from ischemic penumbra by using the Total Protein Extraction Kit according to the manufacturer’s instructions (Catalog No. WLA019, Wanleibio, China). Nuclear NF-κB subunit p65 was extracted using Nuclear and Cytoplasm Protein Extraction kit (Catal. No. WLA020, Wanleibio, China). β-actin and Histone H3 were used as internal reference proteins. Concentrations of protein samples were determined using the BCA method and western blotting was performed as described previously with some modifications. For short, an equal amount of proteins was subjected to 10% sodium dodecyl sulfate polyacrylamide gel electrophoresis (SDS-PAGE). After transferring the proteins onto polyvinylidene difluoride (PVDF) membranes, the membranes were washed with TTBS for 5 min and then incubated with skim milk powder solution for 1 h. Primary antibody against NF-κB subunit p65 (1:500), p-IκBα (1:500), IκBα (1:500), p-ERK (1:500), ERK (1:500), p-JNK (1:500), JNK (1:500), p-p38 (1:500), p38 (1:500), β-actin (1:1000), or Histone H3 (1:500) was added and the membranes were incubated at 4°C overnight. Following washing with TTBS, the membranes were incubated with HRP-conjugated IgG secondary antibodies (1:5000) for 45 min at 37°C. After washing, the blots were developed using Beyo ECL Plus reagent and scanned in the Gel Imaging System. The relative expression levels of the target proteins were calculated with Gel-Pro-Analyzer (Media Cybernetics, USA).

### Statistical Analysis

All the data were expressed in the form of mean ± SD. Multiple comparisons were conducted by Duncan method using general liner model with a significant level of 0.05. All the statistical analysis and image manipulation were conducted using R language version 3.2.1 (R Foundation for Statistical Computing).

## Results

### Administration of GP Alleviated the Impairments on the Cardiac Function and Structure Induced by I/R Injury in Rats

Detail data of LVESP, LVEDP, and FS detection was shown in **Table 1**. I/R injury caused significant reduction of LVESP and FS, but the value of LVEDP was increased post I/R injury induction. Administration of GP significantly alleviated the impairments on cardiac function due to I/R injury. For LVEDP and LVESP, average levels in I/R + GPM and I/R + GPH groups were significantly higher than those in I/R group (*P* < 0.05; **Table 1**). For FS, significant difference was detected only between I/R + GPH and I/R groups (*P* < 0.05; **Table 1**). Moreover, the effect of GP on improving cardiac function was dose-dependent, with GP at 200 mg/kg body weight exhibiting the most powerful treatment effect.

**Table 1:** Treatment with gypenoside (GP) improved the cardiac function in model animals (mean ± SD).

Parameters	Group				
	Sham	I/R	I/R + GPL	I/R + GPM	I/R + GPH
LVESP (mmHg)	128.6 ± 6.8	79.0 ± 6.8^a^	81.3 ± 8.0^a^	93.1 ± 5.1^ab^	102.7 ± 9.0^abc^
LVEDP (mmHg)	6.0 ± 1.9	14.2 ± 3.5^a^	10.9 ± 4.7	8.8 ± 2.2^b^	7.8 ± 1.4^b^
FS (%)	48.3 ± 5.9	33.1 ± 6.3^a^	34.1 ± 4.0^a^	35.5 ± 5.6^a^	45.6 ± 6.0^bc^
LDH (U/L)	967.6 ± 276.5	2921.8 ± 983.8^a^	2208.0 ± 731.7^a^	1994.2 ± 519.1	1477.8 ± 350.4^b^
CK (U/mL)	1.2 ± 0.4	4.7 ± 1.2^a^	3.7 ± 1.5^a^	3.4 ± 1.2^a^	2.2 ± 0.5^b^

*^a^Significantly different from sham group, P < 0.05. ^b^Significantly different from I/R group, P < 0.05. ^c^Significantly different from I/R + GPL group, P < 0.05.*

To confirm the damage to cardiomyocytes due to I/R injury, the levels of LDH and CK in serum samples were also assessed. The statistically significant increases in the content of the two indicators were observed in I/R group compared with sham group (*P* < 0.05; **Table 1**). However, the levels of LDH and CK in GP treated group were markedly decreased, suggesting the potential of GP in protecting cardiac function against I/R injury.

**FIGURE 1:**
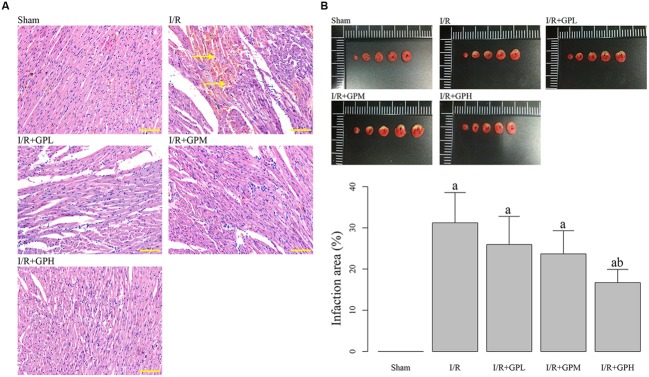
**Administration of gypenoside (GP) alleviated the impairments in myocardial tissues due to I/R injury. (A)** Illustration of hematoxylin and eosin (H&E) staining of the myocardial tissues in different rats groups: the nuclei in myocardial tissue were stained blue and cytoplasm were stained red. Arrows indicating the demolition of cell structure. Scale bar: 100 μm. **(B)** Treatment with GP significantly decrease the infarct area caused by I/R injury. a, significantly different from sham group, *P* < 0.05. b, significantly different from I/R group, *P* < 0.05.

As illustrated by H&E staining, severe damages were observed in I/R group. However, pretreatment with GP obviously ameliorated the damages in myocardial cells (**Figure 1A**). As shown in **Figure 1B**, exposure to GP before I/R injury dramatically decreased the infarction area (*P* < 0.05). However, even for I/R + GPH group, the infarct area was still much larger than that in sham group.

### Administration of GP Increased the Cell Viability in OGD/R Injured H9c2 Cells

**FIGURE 2:**
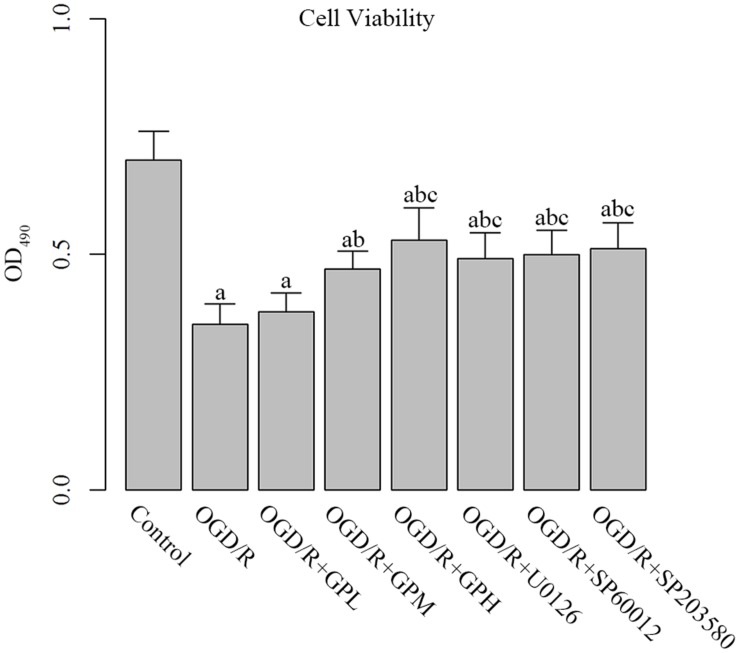
**Administration enhanced cell viability in OGD/R injured H9c2 cells.** a, significantly different from Control group, *P* < 0.05. b, significantly different from OGD/R group, *P* < 0.05. c, significantly different from sham group OGD/R + GPL group, *P* < 0.05.

As shown by MTT assay, OGD/R treatment significantly decreased the cell viability of H9c2 cells compared with control group (*P* < 0.05; **Figure 2**). Administration of GP at different doses all enhanced the cell viability. Additionally, administration of MAPK inhibitors also restored the cell viability of OGD/R injured cells to a similar level of that in OGD/R + GPH group, and no significant difference between the three MAPK inhibitors was detected.

### Administration of GP Inhibited the Production and Nuclear Translocation of NF-κB

**FIGURE 3:**
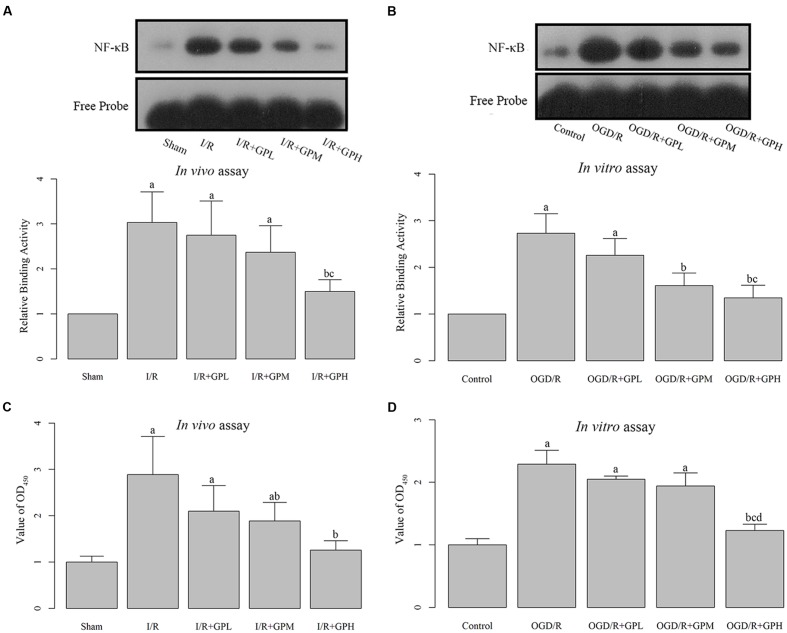
**Treatment with GP significantly inhibited the production and nuclear translocation of NF-κB. (A)** The graph represented the relative NF-κB binding activity, and the image represented the EMSA results in I/R injured rats. **(B)** The graph represented the relative NF-κB binding activity, and the image represented the EMSA results in OGD/R H9c2 cells. **(C)** The NF-κB activity was analyzed using a NF-κB p65 ELISA Kit, and value of OD_450_ represented the activity of NF-κB p65 in I/R injured rats. **(D)** The NF-κB activity was analyzed using a NF-κB p65 ELISA Kit, and value of OD_450_ represented the activity of NF-κB p65 in OGD/R H9c2 cells. a, significantly different from sham or Control group, *P* < 0.05. b, significantly different from I/R or OGD/R group, *P* < 0.05. c, significantly different from sham group I/R + GPL or OGD/R + GPL group, *P* < 0.05. d, significantly different from OGD/R + GPM group, *P* < 0.05.

The DNA binding activity of NF-κB was detected by EMSA. I/R injury induced increase in NF-κB DNA binding activity was significantly inhibited by GP treatment (*P* < 0.05, **Figure 3A**). Moreover, the ELISA assay demonstrated that I/R injury dramatically induced the expression of NF-κB. Administration with GP could suppress the I/R injury-induced NF-κB production (**Figure 3B**). The inhibiting effect of GP on the activation of NF-κB was dose-dependent.

**FIGURE 4:**
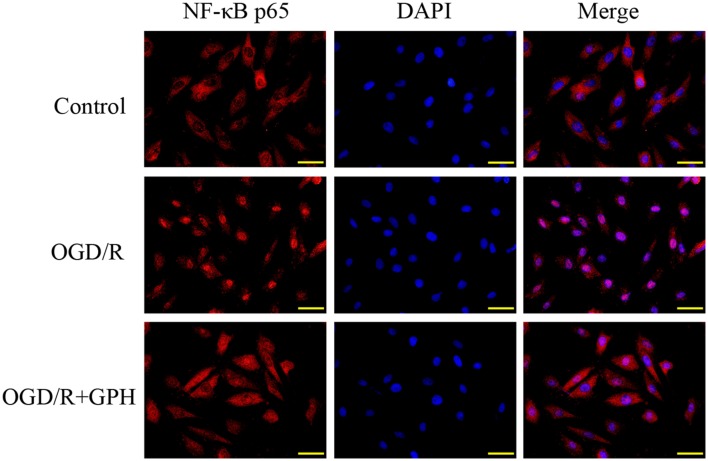
**GP suppressed the nuclear translocation of NF-κB p65 induced by OGD/R in H9c2 cells.** Scale bar: 50 μm.

The changes in DNA-binding activity of NF-κB in H9c2 cells were similar to those in *in vivo* experiments (**Figure 3C**). OGD/R treatment induced the nuclear translocation of NF-κB and GP administration inhibited the DNA-binding activity. Result of ELISA based on H9c2 cells showed a similar pattern to that in *in vivo* assay (**Figure 3D**). Once activated, NF-κB subunit p65 can be translocated into nuclei and bind to its target genes. Thus, immunofluorescence was employed to detect the subcellular localization of NF-κB p65 in H9c2 cells. As shown in **Figure 4**, OGD/R treatment contributed to the nuclear translocation of NF-κB p65 and pre-treatment with GP evidently inhibited the translocation of p65 into nuclei in OGD/R cells.

### Administration of GP Attenuated the Activation of NF-κB via Inhibition of MAPK Pathway

**FIGURE 5:**
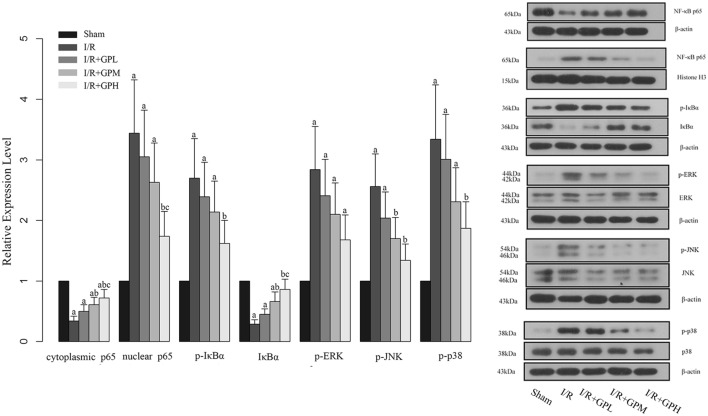
**Effect of GP treatment on the expression of molecules involved in the MAPK mediated NF-κB activation in I/R injured rats.** a, significantly different from sham group, *P* < 0.05. b, significantly different from I/R group, *P* < 0.05. c, significantly different from I/R + GPL group, *P* < 0.05.

We found that I/R injury induced the translocation of p65 into nuclei (**Figure 5**). Consistently, the phosphorylation levels of IκBα, an indicator of NF-κB activation, were enhanced by I/R injury, but the expression of IκBα was decreased by I/R injury (**Figure 5**). These changes were reversed by GP administration. To further evaluate the role of the upstream regulators of NF-κB in the protection of GP against I/R injury, the expression of ERK, p-ERK, JNK, p-JNK, p38, and p-p38 was detected. As shown in **Figure 5**, the phosphorylation status of ERK, JNK, and p38 were induced by I/R injury and pre-treatment with GP inhibited the phosphorylation of the three indicators, suggesting that GP may suppress the activation of NF-κB via inhibition of phosphorylation of ERK, JNK, and p38.

**FIGURE 6:**
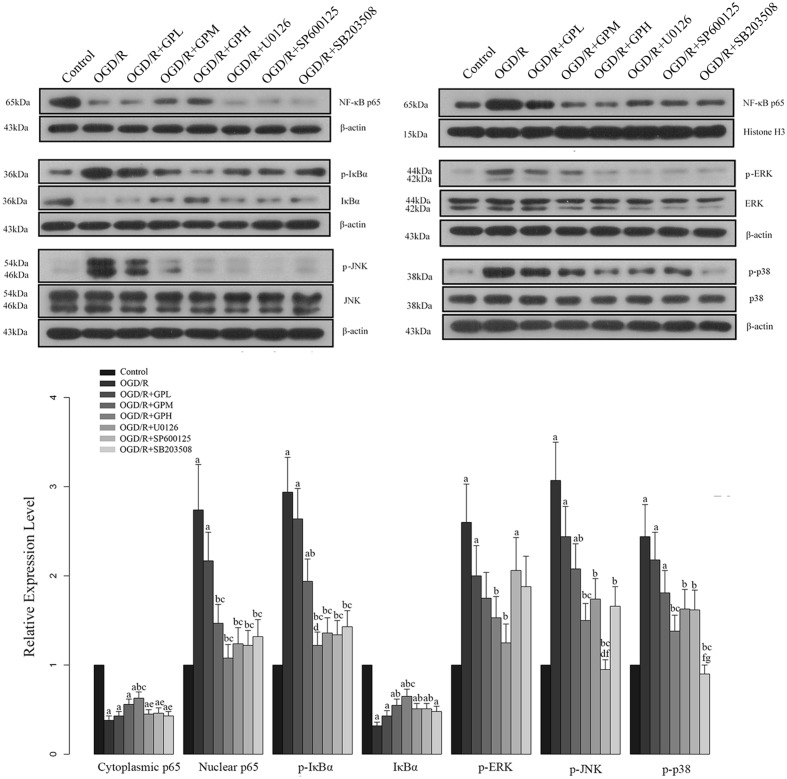
**Effect of GP treatment on the expression of molecules involved in the MAPK mediated NF-κB activation in OGD/R H9c2 cells.** a, significantly different from control group, *P* < 0.05. b, significantly different from OGD/R group, *P* < 0.05. c, significantly different from OGD/R + GPL group, *P* < 0.05. d, significantly different from OGD/R + GPM group, *P* < 0.05. e, significantly different from OGD/R + GPH group, *P* < 0.05. f, significantly different from OGD/R + U0126 group, *P* < 0.05. g, significantly different from OGD/R + SP600125 group, *P* < 0.05.

Based on the results of *in vivo* experiments, western blotting was conducted with three additional groups to further confirm the effects of GP on MAPK mediated NF-κB activation in I/R injured cardiomyocytes. The expression changes of indicators in H9c2 cells were shown in **Figure 6**. The influence of I/R injury on the expression of our targeted molecules was similar to those in *in vivo* experiment and pre-treatment of GP also showed a comparable effect to that in *in vivo* experiments. Additionally, treatment with MAPK pathway inhibitors also attenuated the changes in these molecules induced by OGD/R injury, indicating that GP inhibits NF-κB activation by suppressing the MAPK signaling transduction (**Figure 6**). But the protective effect of GP on cardiomyocytes via MAPK pathway might be more complicated as expected, our results showed that administration with each MAPK inhibitor did not only inhibit the expression of the specific target but also inhibited other MAPK members in OGD/R models (**Figure 6**). In addition, all the three MAPK inhibitors exhibited a similar effect on the activation of NF-κB pathway (**Figure 6**), raising the possibility that the activation of NF-κB by MAPKs might need the actions of multiple pathways.

## Discussion

Definitive therapy for myocardial ischemic disease always depends on the perfusion process, but injury induced by the reperfusion process has casted complex obstacles to the effective treatment of ischemic disease (Ma et al., 2014). Hence, mechanism related to I/R injury has drawn a lot of attention and become a critical subject of cardiovascular studies. Unfortunately, even with plenty of effort being made to improve the myocardial I/R injury recent years, little progress has been achieved. GP is the predominant component of a traditional medicine in Asia. The agent has been reported to have treatment effect on various diseases (Feng et al., 2012; Qin et al., 2012; Zhao et al., 2014). In the present study, GP was employed to ameliorate the I/R injury in rat model and H9c2 cells. In *in vivo* experiments, our data showed that values of LVESP, LVEDP, and FS were improved by pre-treatment of GP. Moreover, the production of indicators for necrotic damage and cardiomyocyte injury, including LDH and CK, were measured and the results confirmed protective effect of GP on cardiac function against I/R injury. GP administration also alleviated the damage to cardiomyocyte structure. Based on the results of H&E and TTC staining, deterioration in cardiomyocytes and infarction in myocardial tissues were reduced by precondition of GP.

As mentioned above, I/R injury is characterized by the release of many cytokines and chemokines (Nakamura et al., 2000; Won et al., 2002; Ekshyyan and Aw, 2004; Xiao et al., 2008), expressions of which always result in the activation of NF-κB (Chung et al., 2012). The molecule regulates the expression of variable immediate-early and stress response genes (Frantz et al., 2007) and plays a decisive role in I/R injuries. Numerous studies have assessed the potency of targeting NF-κB pathway as a therapy for I/R injury prevention and treatment (Frantz et al., 2007; Kim et al., 2009; Hamid et al., 2010; Ma et al., 2014). In the current study, it was showed that GP pre-treatment could reduce the phosphorylation level of IκBα as well as inhibiting the translocation of NF-κB p65 to nuclei. Simultaneously, incubation with GP also increased the content of IκBα in H9c2 cells, which would contribute to the inhibition of NF-κB activity. It is widely recognized that blockade of NF-κB pathway can lead to the reduced release of pro-inflammatory cytokines and chemokines (Onai et al., 2004; Kim et al., 2010; Gu et al., 2012; Ramachandran et al., 2012). And disruption of NF-κB’s translocation in nuclei will weaken the synthesis of monocyte chemoattractant protein-1 (MCP-1) during chronic inflammation (Higa et al., 2012), reduce the expression of IL-6 (Lee et al., 2013), and decrease the secretion of MMP-2 and MMP-9 (Lu and Lai, 2013). This regulated pattern of NF-κB pathway will contribute to the resistance of cells to the degradation of the extracellular matrix and inflammatory response. Combined with these studies, our data evidently showed that GP had the capability to protect cardiomyocytes against I/R injury through the inhibition of NF-κB pathway.

The three members of MAPK pathway, ERK, JNK, and p38 are capable of initiating the activity of NF-κB (Ma et al., 2014). In the current study, it was of interest to note that the ERK, JNK, and p38 were all activated simultaneously in cardiomyocytes when exposed to I/R manipulation. The temporal expression profile as well as the extent of activation of the three MAPK members was similar, suggesting that their activation is a common response of the cardiomyocytes to I/R. However, the phosphorylation levels of ERK and p38 in the present study were quite different from that in Ma et al.’s (2014) study, in which the levels of p-ERK and p-p38 did not change after ginsenoside Rb3 treatment. Ma et al. (2014) had inferred that regulation of NF-κB was cell or tissue specific and effect of natural products administration depended on the cell sensitivity to the certain agent. Given that p-ERK and p-p38 being down-regulated by GP in the present study, GP should have a distinct mechanism in protecting myocytes against I/R injury as compared with commonly recognized ginsenoside Rb3.

To dissect the roles of the three indicators in I/R induced NF-κB activation, U0126, SP600125, and SB203580 were employed in *in vitro* assay. Based on the western blotting assay, it was not surprising to find that the three inhibitors all suppressed the expression of their specific targets in a similar pattern to that of GP. However, the effect of each inhibitor had more impact on its specific MAPK, it seemed that they also inhibited other MAPKs. In the study of Yue et al. (2000) based on cardiomyocytes I/R injury, MEK1/MEK2 inhibitor PD98059 at 50 μmol/L significantly enhanced the activity of p38 and JNK while the effect of SB203580 on JNK varied with concentration. Thus, some researchers concluded that the roles of ERK, JNK, and p38 are cell type specific (Liu et al., 1996; Nemoto et al., 1998). Taken the performance of the inhibitors in our study into consideration, the cross talk between members of MAPK pathway is extremely complicated. Even though the inhibitors employed in the current study are highly specific, regulation of one member in MAPK pathway will indirectly influence the activity of other MAPKs.

Although numerous studies have reported that activation of ERK, JNK, and p38 contribute to the up-regulation of NF-κB pathway, few ones have provide a detail explanation on the regulation scheme of NF-κB by MAPKs. Some researchers infer that ERK, JNK, and p38 can increase the phosphorylation of IKK-β which is responsible for IκBα degradation and NF-κB nuclear translocation (Huang, 2008; Yeh et al., 2011; Slomiany and Slomiany, 2013). Additionally, other work with some insights into the subject indicates that: ghrelin, a peptide hormone, is capable of suppressing IKK-β in an anti-p38/JNK manner. However, the modulatory effect of the agent is also associated with an enhancement in ERK activity (Slomiany and Slomiany, 2013). Concatenated with our finding that each MAPK inhibitor exhibited a similar regulation pattern on NF-κB, it is hypothesized that the activation of NF-κB may require involvement of the three MAPKs at the same time. To test the hypothesis, further work regarding the mechanism of GP in protecting cardiomyocytes against I/R injury needs to be conducted in the future.

## Conclusion

The major findings outlined in the current study indicated that GP regulated I/R injury via the following mechanism: (1) GP contributed to the blockade of NF-κB p65 translocation into nuclei, which would in turn inhibit NF-κB’s binding to certain transcriptional region of pro-inflammatory factors. (2) GP administration would decrease the phosphorylation of IκBα and increase the level of IκBα in cardiomyocytes, which would inhibit the activation of NF-κB as well. (3) GP also exerted its function by inhibiting the expression of ERK, JNK, and p38, which would negative regulate the activation NF-κB. Our study evidently showed the potential of GP to be a promising agent to prevent cardiomyocytes against I/R injury. However, shortcomings still exist in the current study, our experiments were designed to reveal the effect and to preliminarily uncover the pathway through which GP takes action on I/R injury, but we ignored to determine the suitable practical dose or the effect of GP comparable to current treatment modalities. To uncover the underlying mechanism involved in the treatment effect of GP and facilitate the application of the agent, more comprehensive work is being conducted in our lab.

## Author Contributions

HY substantially contributed to the conception or design of the work and drafting of the work and final approval of the version to be published; LS substantially contributed to the acquisition of data for the work and drafting of the work; GQ substantially contributed the acquisition, analysis, or interpretation of data for the work; SZ substantially contributed the analysis and interpretation of data for the work; YG substantially contributed the analysis and interpretation of data for the work; YL substantially contributed to the conception or design of the work and drafting of the work.

## Conflict of Interest Statement

The authors declare that the research was conducted in the absence of any commercial or financial relationships that could be construed as a potential conflict of interest.

## Supplementary material

FIGURE S1 **General structure for dammarane-type gypenosides (where R1 ¼ glucose, rhamnose; R2 ¼ glucose, rhamnose, etc.; R3 ¼ CH3, CH2OH, CHO; R4 ¼ H, CH2OH; and R5 ¼ glucose, xylose, etc.)**.
